# Racial/ethnic differences in the clinical presentation and survival of breast cancer by subtype

**DOI:** 10.3389/fonc.2024.1443399

**Published:** 2024-08-16

**Authors:** Vutha Nhim, Alfonso E. Bencomo-Alvarez, Luis Alvarado, Michelle Kilcoyne, Mayra A. Gonzalez-Henry, Idaly M. Olivas, Mehrshad Keivan, Sumit Gaur, Zuber D. Mulla, Alok K. Dwivedi, Shrikanth S. Gadad, Anna M. Eiring

**Affiliations:** ^1^ Paul L. Foster School of Medicine, Texas Tech University Health Sciences Center El Paso, El Paso, TX, United States; ^2^ University of Arkansas for Medical Sciences, Washington Regional Medical Center, Fayetteville, AR, United States; ^3^ Center of Emphasis in Cancer, Department of Molecular and Translational Medicine, Texas Tech University Health Sciences Center El Paso, El Paso, TX, United States; ^4^ St. Jude Children’s Research Hospital, Memphis, TN, United States; ^5^ Biostatistics and Epidemiology Consulting Lab, Office of Research, Texas Tech University Health Sciences Center El Paso, El Paso, TX, United States; ^6^ Baylor College of Medicine, Houston, TX, United States; ^7^ Burrell College of Osteopathic Medicine, Las Cruces, NM, United States; ^8^ Department of Internal Medicine, Texas Tech University Health Sciences Center El Paso, El Paso, TX, United States; ^9^ Department of Obstetrics and Gynecology, Texas Tech University Health Sciences Center El Paso, El Paso, TX, United States; ^10^ Office of Faculty Development, Texas Tech University Health Sciences Center El Paso, El Paso, TX, United States; ^11^ Julia Jones Matthews School of Population and Public Health, Texas Tech University Health Sciences Center, Abilene, TX, United States

**Keywords:** race/ethnicity, breast cancer (BC), United States/Mexico border, population-based study, cancer health disparities

## Abstract

**Background:**

Breast cancer (BC) affects racial and ethnic groups differently, leading to disparities in clinical presentation and outcomes. It is unclear how Hispanic ethnicity affects BC outcomes based on geographic location and proximity to the United States (U.S.)/Mexico border. We hypothesized that the impact of race/ethnicity on BC outcomes depends on geographic location and country of origin within each BC subtype.

**Methods:**

We analyzed BC data from the Texas Cancer Registry by race/ethnicity/birthplace according to BC subtype (luminal A/luminal B/human epidermal growth factor receptor 2 [HER2]/triple-negative breast cancer[TNBC]). Other covariates included age, geographic location (U.S., Mexico), residency (border, non-border), treatments, and comorbidities. Crude and adjusted effects of race/ethnicity and birthplace on overall survival (OS) were analyzed using Cox regression methods.

**Results:**

Our analysis of 76,310 patient records with specific BC subtypes revealed that Hispanic and non-Hispanic Black (NHB) patients were diagnosed at a younger age compared with non-Hispanic White (NHW) patients for all BC subtypes. For the 19,748 BC patients with complete data on race/ethnicity/birthplace/residency, Hispanic patients had a higher mortality risk in the Luminal A subtype, regardless of birthplace, whereas U.S.-born Hispanics had a higher risk of death in the TNBC subtype. In contrast, NHB patients had a higher mortality risk in the Luminal A and HER2 subtypes. Residence along the U.S./Mexico border had little impact on OS, with better outcomes in Luminal A patients and worse outcomes in Luminal B patients aged 60–74 years.

**Conclusion:**

Race/ethnicity, geographic birth location, and residency were significant predictors of survival in BC. Migration, acculturation, and reduced healthcare access may contribute to outcome differences.

## 1 Introduction

In 2020, breast cancer (BC) became the leading cause of global cancer incidence, overtaking lung cancer with 2.3 million new cases and ranking as the fifth cause of death worldwide (1). Among women in the United States (U.S.), BC is the most frequently diagnosed cancer and remains the second leading cause of cancer-related deaths, with 297,790 new cases and 43,700 deaths in 2023 alone (2). Developing countries exhibit higher BC mortality rates relative to their developed counterparts despite having lower incidence rates, which is likely due to reduced screening measures (3, 4). Despite a decade of declining BC mortality rates, disparities persist among racial and ethnic minorities in the U.S. The BC mortality rate has dropped more slowly for Hispanics than non-Hispanic White (NHW) women (5), and racial/ethnic groups are more frequently diagnosed with advanced-stage tumors due to lower utilization of mammography services and delays in follow-up care (6, 7). Moreover, foreign-born Hispanic patients had lower incidence and mortality rates compared with U.S.-born Hispanics (5). Whereas Hispanic women in the U.S. have demonstrated lower BC incidence and mortality rates compared with NHW and non-Hispanic Black (NHB) women (8), BC remains the primary cause of cancer-related deaths for female Hispanics. Similarly, NHB women have a 4% lower BC incidence rate compared with NHW women, but face a 40% higher chance of death (9). In fact, aggressive BC subtypes like triple-negative breast cancer (TNBC) are more prevalent in non-White populations (10, 11). New strategies and treatments will be required to improve outcomes for minority populations with BC.

BC is a clinically and genetically heterogeneous disease characterized by multiple different subtypes. Molecular subtyping has provided a valuable tool for predicting clinical outcomes in BC (12). Thus, the BC subtype is an important factor that needs to be accounted for in the analysis of overall survival (OS). Breast cancer (BC) can be classified into four major subtypes based on hormone receptor (HR) status: estrogen receptor (ER), progesterone receptor (PR), and HER2. The subtypes are Luminal A (HR+/HER2-), Luminal B (HR+/HER2+), HER2+/ER-/PR-, and triple-negative (HR-/HER2-) (13–16). Among these subtypes, Luminal A has the best prognosis, followed by Luminal B (15, 16). On the other hand, TNBCs are associated with the worst prognosis (16, 17). HER2-positive can be diagnosed at a younger age compared with Luminal A (15, 18). TNBCs are more prevalent among Hispanic women compared with NHW women, and they are also common among NHB women. Luminal A is more common among white women (19–21). The high prevalence of TNBC among NHB and Hispanic women could be the reason for the poor prognosis in these populations (2, 22–24).

There is a dearth of information available about cancer rates along the U.S./Mexico border region. One study reported increased cancer mortality among younger Hispanic Americans in the U.S./Mexico border region spanning California, Arizona, New Mexico, and Texas (25). Similarly, using data from the Texas Cancer Registry (TCR), we demonstrated that Hispanic patients with certain hematologic malignancies located along the Texas/Chihuahua border (Health Service Region 10 [HSR10]) were diagnosed at younger ages, presented with increased numbers of comorbidities, and had worse OS compared with the rest of Texas (26). Although racial and ethnic disparities persist in the presentation and outcomes of BC, the influence of race and ethnicity on survival status in the different BC subtypes remains unclear. Moreover, the impact of Hispanic ethnicity on BC outcomes concerning birth country and proximity to the U.S./Mexico border requires further examination, especially within each BC subtype. Previous studies of the border region have reported higher BC presentation and mortality rates for women who grew up in the U.S. or immigrated at a young age compared with Mexican Hispanics or those who immigrated at older ages (27). In addition, a link between diabetes, obesity, and BC health disparities among Hispanic women has been established (28). Hispanic and Black women have a higher prevalence of obesity (29, 30), but their comorbidities and biomarkers can be completely different (30–33). Weight gain could be associated with Hispanics compared to other ethnicities (28, 30), negatively affecting metabolic-related disorder outcomes (30, 34); due to this, insulin resistance and metabolic syndrome are common among Hispanics (30, 35, 36), which is linked to increased aggressive BC subtypes (30, 37, 38). Obesity and other comorbidities can affect treatment (30, 39–41), but not much is known about their contribution among Hispanics. Quality of life substantially reduces post-diagnosis among Hispanics (30, 42, 43). Moreover, obesity can impact the dosages required for therapeutic intervention (30, 44, 45), and can also affect surgery, radiotherapy, and adjuvant chemotherapy (30, 46–49).

In the present study, we aimed to evaluate differences in BC presentation and survival between NHW, NHB, and Hispanic patients (born in Mexico versus the U.S.), and comparing the border region with the rest of Texas. We also assessed covariates associated with mortality in BC subtypes throughout Texas. We hypothesized that the impact of race/ethnicity on BC outcomes depends on a combination of BC subtype, country of origin (U.S. vs. Mexico), and residency (HSR10 vs. rest of Texas). Specifically, we sought to understand which BC subtypes have poor presentation and worse OS among Mexican Hispanics compared with non-Hispanics. Additionally, we wanted to pinpoint the BC subtypes demonstrating worse outcomes in the border region to prioritize appropriate screening measures and treatment for these patients.

## 2 Methods

### 2.1 Data sources and study population

**Figure 1:**
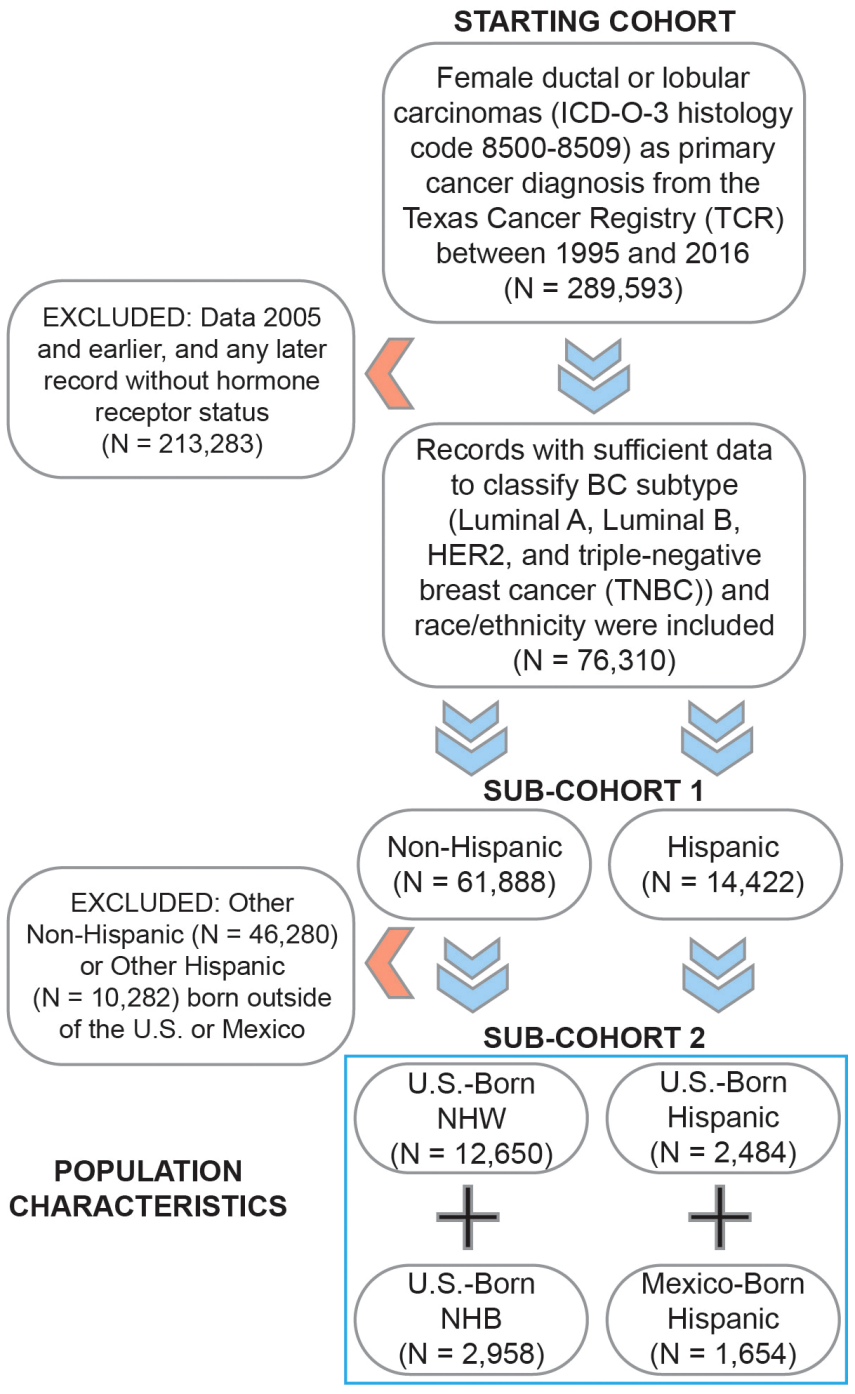
Sample selection diagram and patient characteristics for the current study. The selection of breast cancers from the Texas Cancer Registry was performed using the International Classification of Diseases for Oncology third edition (ICD-O-3) histology codes for the following diseases: ductal and lobular carcinomas, followed by stratification into ER+, PR+, HER2+, and TNBC subtypes.

BC data (International Classification of Disease: ICD-O-3 histology codes 8500–8509) were obtained from the Texas Cancer Registry (TCR), which serves as a statewide population-based registry of cancer prevalence and burden in Texas (https://www.dshs.texas.gov/tcr/). All breast cancer data available (i.e., 1995 to 2016) from the TCR were reviewed to understand the clinical presentation and survival of BC for NHW, NHB, and Hispanic patients, as well as the population at the Texas/Chihuahua border in HSR10. HSR10 refers to communities at the U.S./Mexico border near Chihuahua, Mexico. The region is medically underserved, with significant barriers to healthcare access (50). Hispanics in our dataset were divided by birth country (Mexico or U.S.). The following BC subtypes were analyzed: Luminal A (estrogen and/or progesterone receptor-positive (ER+ or PR+) and human epidermal growth factor receptor 2-negative (HER2-)); Luminal B (ER+ and/or PR+ and HER2+), HER2 positive (ER-, PR-, and HER2+), and TNBC (ER-, PR- and HER2-). We included only female BC patients with sufficient data to classify their BC subtypes, called sub-cohort 1. In secondary criteria, we only included BC patients from sub-cohort 1 with appropriate race/ethnicity/birthplace/age classifications, called sub-cohort 2. This study was approved by the Institutional Review Boards at Texas Tech University Health Sciences Center El Paso (E19045) and the TCR (19–006). **Figure 1** illustrates the data selection process. The study sample included 289,593 female BC records from the TCR. BC data not having complete hormone receptor information (213,283 records) were excluded from the study. Baseline characteristics for the excluded BC patients are available in **Supplementary Table S1**. The remaining 76,310 records (sub-cohort 1) had a specific classification by subtype (**Table 1** and **Supplementary Table S2**). In contrast to data from the Surveillance, Epidemiology, and End Results (SEER) Program, entries without a reported ethnicity in the TCR are not assumed to be non-Hispanic but instead coded as unknown, thereby reducing the potential for perceived bias from self-reporting of ethnicity (51). Patients identified as Other non-Hispanic (46,280) or Other Hispanic (10,282) who were not born in the U.S. or Mexico were excluded from survival calculations due to a lack of confidence surrounding ethnicity. In total, 19,748 records (sub-cohort 2) had complete information regarding classification by race, ethnicity, BC subtype, and birthplace (**Table 2** and **Supplementary Table S3**).

**Table 1:** Breast cancer distribution of sub-cohort 1 (76,310 breast cancer patients from the Texas Cancer Registry from 1995–2016) according to subtype, based on age, race, ethnicity, geographic location, and socioeconomic indicators.

Subtype (Sub-Cohort 1)	Luminal A	Luminal B	HER2	TNBC
N (%)	53,777 (100)	8,790 (100)	3,699 (100)	10,044 (100)
Age Ranges				
18-39	2,237 (4.2)	847 (9.6)	332 (9.0)	941 (9.4)
40-59	20,704 (38.5)	4,086 (46.5)	1,830 (49.5)	4,636 (46.2)
60-74	21,927 (40.8)	2,875 (32.7)	1,153 (31.2)	3,291 (32.8)
75+	8,909 (16.6)	982 (11.2)	384 (10.4)	1,176 (11.7)
Race & Ethnicity				
Hispanic	9,504 (17.7)	1,965 (22.4)	869 (23.5)	2,084 (20.7)
NHW	36,861 (68.5)	5,367 (61.0)	2,136 (57.7)	5,470 (54.5)
NHB	5,606 (10.4)	1,090 (12.4)	510 (13.8)	2,166 (21.6)
HSR				
Rest of Texas	52,303 (97.3)	8,452 (96.2)	3,563 (96.3)	9,691 (96.5)
HSR 10	1,474 (2.7)	338 (3.8)	136 (3.7)	353 (3.5)
Country of Birth				
U.S.	12,292 (22.9)	2,103 (23.9)	927 (25.1)	2,983 (29.7)
Mexico	991 (1.8)	271 (3.1)	130 (3.5)	341 (3.4)
Ethnicity/Race & Birthplace				
Hispanic Born in U.S.	1,567 (2.9)	333 (3.8)	149 (4.0)	435 (4.3)
Hispanic Born in Mexico	947 (1.8)	260 (3.0)	125 (3.4)	324 (3.2)
NHW Born in U.S.	8,913 (16.6)	1,389 (15.8)	617 (16.7)	1,731 (17.2)
NHB Born in U.S.	1,671 (3.1)	341 (3.9)	154 (4.2)	792 (7.9)
Hispanic Other	6,990 (13.0)	1,372 (15.6)	595 (16.1)	1,325 (13.2)
Non-Hispanic Other	33,689 (62.6)	5,095 (58.0)	2,059 (55.7)	5,437 (54.1)
Ethnicity/Race HSR				
Hispanic HSR 10	1,092 (2.0)	254 (2.9)	109 (2.9)	277 (2.8)
Hispanic - Rest of Texas	8,412 (15.6)	1,711 (19.5)	760 (20.5)	1,807 (18.0)
Non-Hispanic	42,467 (79.0)	6,457 (73.5)	2,646 (71.5)	7,636 (76.0)
Urbanization				
Rural	468 (0.9)	74 (0.8)	37 (1.0)	57 (0.6)
Semi-Urban	5,697 (10.6)	967 (11.0)	348 (9.4)	980 (9.8)
Urban	47,583 (88.5)	7,743 (88.1)	3,312 (89.5)	9,003 (89.6)
Primary Insurance				
Uninsured	2,723 (5.1)	649 (7.4)	324 (8.8)	819 (8.2)
Private Insurance	25,492 (47.4)	4,609 (52.4)	1,896 (51.3)	5,007 (49.9)
Medicaid/Medicare	23,292 (43.3)	3,122 (35.5)	1,301 (35.2)	3,782 (37.7)
Poverty Level				
No	40,236 (74.8)	6,416 (73.0)	2,635 (71.2)	6,984 (69.5)
Yes	12,998 (24.2)	2,303 (26.2)	1,035 (28.0)	2,979 (29.7)

HER2, human epidermal growth factor 2; HSR, health service region; NHB, non-Hispanic Black; NHW, non-Hispanic White; TNBC, triple-negative breast cancer; U.S., United States.

**Table 2:** Sub-cohort 2 consisted of 19,748 patients with complete data on breast cancer subtype, race, ethnicity, birthplace, and age.

	Mexico-Born Hispanic	U.S.-Born Hispanic	NHW	NHB	P
BC Subtype (Sub-Cohort 2) Number of Patients					
**N (%)**	1,656 (100)	2,484 (100)	12,650 (100)	2,958 (100)	<0.0001*
**Luminal A**	947 (57.2)	1,567 (63.1)	8,913 (70.5)	1,671 (56.5)	
**Luminal B**	260 (15.7)	333 (13.4)	1,389 (11.0)	341 (11.5)	
**HER2**	125 (7.5)	149 (6.0)	617 (4.9)	154 (5.2)	
**TNBC**	324 (19.6)	435 (17.5)	1,731 (13.7)	792 (26.8)	
Average ± SD Age in Years					
**Luminal A**	57.4 ± 13.8	60.9 ± 14.0	65.1 ± 13.0	60.4 ± 13.7	0.0001
**Luminal B**	54.4 ± 12.8	57.3 ± 15.0	62.3 ± 14.4	57.6 ± 13.8	0.0001
**HER2**	54.6 ± 12.5	58.1 ± 13.6	61.0 ± 13.7	58.4 ± 11.6	0.0001
**TNBC**	52.2 ± 12.9	54.5 ± 14.9	61.5 ± 14.2	56.3 ± 12.8	0.0001

(Top) Number of breast cancer patients based on subtype and race. *The p-value was calculated from a 4 x 4 chi-square test. (Bottom) The average age of breast cancer diagnosis stratified by subtype and race/ethnicity. Age distributions were compared across race/ethnicity within each subtype using one-way analysis of variance. HER2, human epidermal growth factor 2; NHB, non-Hispanic Black; NHW, non-Hispanic White; SD, standard deviation; TNBC, triple-negative breast cancer; U.S., United States.

### 2.2 Primary exposures and outcomes

OS for each BC subtype was the primary outcome, with race/ethnicity, birthplace (U.S.-born NHW, U.S.-born NHB, U.S.-born Hispanic, Mexico-born Hispanic), geographic location (HSR10, rest of Texas), and residency (HSR10, rest of Texas) as the independent variables. OS was measured from the year of diagnosis to the year of vital status. Patients without a date of death or last contact but were alive after December 2016 were assumed to be alive.

### 2.3 Covariates

The following covariates were considered as possible confounders of the relationship between race/ethnicity and OS for patients with BC: age (18–39, 40–59, 60–74, 75+ years), grade (I, II, III, IV), SEER stage (localized, regional, distant), treatment (hormone therapy, chemotherapy, radiation therapy, mastectomy, distal lymph or tissue/organ surgery), urbanization (rural, semi-urban, urban), and number of comorbidities (1–2, 3–4, 5–6, 7–8, 9–10). We chose not to control for measures of poverty and stage at diagnosis since these variables are on the causal pathway between our exposure variable (race/ethnicity) and the outcome (time between cancer diagnosis and death) (52). Controlling for causal intermediates using standard regression methods results in biased estimates of the total effect of race/ethnicity (52, 53). Since race and ethnicity are closely linked to socioeconomic status (SES) (52), poverty indicators and primary payer at diagnosis were excluded from the analysis. Based on the literature, Hispanic patients are often diagnosed with BC at a younger age, which likely affects OS. To investigate the potential impact of age on survival, patients were categorized into four age groups (in years) for each BC subtype: 18–39, 40–59, 60–74, and ≥75. Differences in OS among NHW, NHB, and Hispanic patients due to treatment effects were evaluated by stratifying patients based on their intervention(s).

### 2.4 Statistical analyses

We summarized continuous variables with mean and standard deviation (SD), whereas categorical variables were summarized with frequency and percentage. All covariates were summarized in frequencies with percentages by BC subtypes. The average age of patients was compared between race/ethnicity by one-way analysis of variance within each BC subtype. All the categorical variables were compared by race/ethnicity/country of birth using a chi-square test. The association of each exposure and covariate with OS in each BC diagnosis was determined using a Cox proportional hazards regression analysis. Multivariable Cox proportional hazards regression was used to determine the adjusted association between primary exposures and OS for each BC diagnosis. The multivariable analysis included all the relevant covariates simultaneously in the analysis. *A priori*, critical covariates were adjusted regardless of their significance level in the multivariable analyses as per the study objectives (54). The results of Cox models were summarized with hazard ratio (HR) and 95% confidence interval (CI). All hazard ratio (HR) calculations in the multivariable analysis were adjusted for the remaining variables listed in the table. The assumption of proportional hazards was tested by inspecting log-log plots for each predictor variable. Severe violations (as indicated by non-parallel lines) were not detected. Kaplan-Meier curves were constructed to describe survival for Hispanic and non-Hispanic patients according to diagnosis or regional location and were compared using log-rank tests. P-values less than or equal to 5% were considered statistically significant results. All the statistical analyses were conducted using SAS version 7 (SAS Institute Inc., Cary, NC, USA) and followed the statistical analysis and reporting checklists (55).

## 3 Results

### 3.1 Cohort selection

The total number of BC patients in our cohort (sub-cohort 1) included 53,777 Luminal A patients (70.5%), 8,790 Luminal B patients (11.5%), 3,699 HER2 patients (4.8%), and 10,044 TNBC patients (13.2%) (**Table 1**). For survival calculations, patients who did not identify as NHW, NHB, or Hispanic born in the U.S. or Mexico (Other) were excluded from the analysis (56,562). Of the remaining 19,748 BC patients (sub-cohort 2), 12,650 (64.1%) identified as NHW, 2,958 (15.0%) identified as NHB, 2,484 (12.6%) identified as U.S.-born Hispanic, and 1,656 (8.4%) identified as Mexico-born Hispanic (**Table 2** and **Figure 1**). There were no differences in reporting standards by ethnicity, as 47.5% of non-Hispanics and 47.8% of Hispanics had a specific diagnosis (not shown).

### 3.2 Breast cancer presentation by BC subtypes

**Table 1** shows the distribution of patients with known BC subtypes based on age, race, ethnicity, geographic location, and socioeconomic indicators. The Luminal A subtype of BC was more common in patients older than 60 years, whereas the other BC subtypes were more common in patients 40–59 years of age. NHW patients were more likely to be diagnosed with the Luminal A or Luminal B subtypes of BC. Hispanic patients were more likely to be diagnosed with the HER2 subtype, whereas NHB patients were more likely to be diagnosed with TNBC (**Table 1**). Proportionally, Hispanics born in the U.S. had higher rates of all BC subtypes than those born in Mexico. Overall, about half of the patients had private insurance; however, many patients were covered by Medicaid/Medicare. Finally, the highest poverty rate was observed in TNBC patients (29.7%) (**Table 1**).

**Supplementary Table S2** illustrates patient distribution between subtypes based on tumor grade, treatment, and comorbidity burden. TNBC patients had the most significant proportion of patients diagnosed at grade III/IV (74.9%), followed by HER2 (69.0%), Luminal B (47.4%) and Luminal A (19.8%). However, HER2 had the most significant proportion of patients with regional and distant metastasis (50.1%). Over half of the patients had no comorbidities across all subtypes. However, the comorbidities most highly associated with all BC subtypes were cardiovascular diseases, endocrine/nutritional/metabolic/immune disorders, and genitourinary diseases.

### 3.3 Breast cancer presentation by race/ethnicity and country of birth

Most Hispanic patients developed BC between 40 and 59 years of age, regardless of birthplace (**Supplementary Table S3**). However, in that age group, Hispanic patients born in Mexico developed proportionally more BC than those born in the U.S. (55.8% versus 41.0%). In contrast, NHW patients developed BC older than 60 years (63.9%). Many Hispanic patients resided in HSR10 compared with other racial/ethnic groups (9.3% and 24.3% for Hispanics born in the U.S. and Mexico, respectively). The bottom of **Table 2** shows the age at diagnosis for each disease, comparing NHW, NHB, and Hispanic BC patients throughout Texas. NHB and Hispanic patients in Texas were diagnosed at younger ages than NHW patients for all diseases analyzed (P<0.0001). When we compared the distribution of BC among Hispanics born in the U.S. versus Mexico, Hispanics born in Mexico developed BC at younger ages (**Table 2**). As expected, Hispanics born in Mexico had the highest prevalence of uninsured patients (42.7%), while NHW had the highest proportion of private insurance coverage (42.4%, **Supplementary Table S3**). Most patients born in the U.S. were enrolled in Medicaid/Medicare. Finally, 53.3% of Hispanic patients born in Mexico were classified as impoverished, followed by NHB (42.9%), Hispanics born in the U.S. (42.3%), and NHW (15.3%) (**Supplementary Table S3**).

**Table 3** describes the tumor grade, treatment, and comorbidity distribution for U.S.-born Hispanics, Mexico-born Hispanics, NHW, and NHB patients. NHB patients had the highest percentages of grade III/IV tumors (50.9%) with distant metastasis (17.4%), followed by Hispanics born in Mexico (42.8% and 15.8%), Hispanics born in the U.S. (39.5% and 12.6%), and NHW (32.8% and 10.1%). Similar comorbidities were noted across races and ethnicities, except for Hispanics born in Mexico, who presented with higher rates of 1–2 or 3–4 comorbidities (**Table 3**). Hispanics born in the U.S. had the highest prevalence of endocrine, nutritional, metabolic, or immune disorders (24.5%), followed by NHB (23.9%), Hispanics born in Mexico (23.6%), and NHW (18.6%). NHBs were most likely to be observed with cardiovascular diseases (30.2%), followed by Hispanic patients born in the U.S. (24.7%), Hispanic patients born in Mexico (22.7%), and NHW (21.2%). Hispanics born in Mexico had a higher proportion of genitourinary comorbidities (17.3%), followed by NHB (14.3%), Hispanics born in the U.S. (11.6%), and NHW (10.6%). Finally, NHB patients had the highest rate of mortalities (48.9%), followed by Hispanics born in the U.S. (40.2%), NHW (39.0%), and Hispanics born in Mexico (35.8%).

**Table 3:** Demographic and socioeconomic characteristics of sub-cohort 2 (19,748 breast cancer patients with complete data on subtype/race/ethnicity/birthplace/age) based on tumor grade, treatment, and comorbidity burden.

Race/Ethnicity (Sub-Cohort 2)	Mexico-Born Hispanic	U.S.-Born Hispanic	NHW	NHB	P
N (%)	1,656 (100)	2,484 (100)	12,650 (100)	2,958 (100)	
**Grade**					<0.001
Grade-I	151 (9.1)	331 (13.3)	2,353 (18.6)	306 (11.5)	
Grade-II	511 (30.9)	860 (34.6)	5,077 (40.1)	899 (30.4)	
Grade-III	708 (42.8)	981 (39.5)	4,129 (32.6)	1,493 (50.5)	
Grade-IV	7 (0.004)	8 (0.003)	26 (0.2)	13 (0.4)	
**SEER Stage**					<0.001
Localized	657 (39.7)	1,253 (50.4)	7,231 (57.2)	1,269 (42.9)	
Regional	702 (42.4)	862 (34.7)	3,880 (30.7)	1,104 (37.3)	
Distant	261 (15.8)	312 (12.6)	1,282 (10.1)	515 (17.4)	
**Hormone Therapy**					<0.001
Yes	388 (23.4)	533 (21.5)	3,305 (26.1)	620 (21.0)	
**Chemotherapy**					<0.001
Yes	784 (47.3)	941 (37.9)	3,869 (30.6)	1,184 (40.0)	
**Radiation Therapy**					<0.001
Yes	332 (20.0)	480 (19.3)	2,750 (21.7)	535 (18.1)	
**Mastectomy**					<0.001
Yes	911 (55.0)	1,496 (60.2)	7,999 (63.2)	1,486 (50.2)	
**Distal Lymph or Tissue/Organ Surgery**					<0.001
Yes	28 (1.7)	54 (2.1)	320 (2.5)	73 (2.5)	
**Number of Comorbidities**					<0.001
None	755 (45.6)	1,262 (50.8)	6,887 (54.4)	1,344 (45.4)	
1-2	391 (23.6)	473 (19.0)	2,251 (17.8)	570 (19.3)	
3-4	225 (13.6)	284 (11.4)	1,302 (10.3)	371 (12.5)	
5-6	84 (5.1)	161 (6.5)	705 (5.6)	222 (7.5)	
7-8	41 (2.5)	77 (3.1)	349 (2.8)	113 (3.8)	
9-10	43 (2.6)	95 (3.8)	365 (2.9)	135 (4.6)	
**Infectious Diseases**					<0.001
Yes	28 (1.7)	58 (2.3)	177 (1.4)	66 (2.2)	
**Endocrine, Nutritional, Metabolic, Immune Disorders**					<0.001
Yes	391 (23.6)	609 (24.5)	2,355 (18.6)	707 (23.9)	
**Blood and Blood Forming Organ Disorders**					<0.001
Yes	68 (4.1)	114 (4.6)	367 (2.9)	186 (6.3)	
**Mental Disorders**					<0.001
Yes	96 (5.8)	212 (8.5)	1,041 (8.2)	300 (10.1)	
**Neurological Diseases**					<0.001
Yes	68 (4.1)	120 (4.8)	614 (4.9)	165 (5.6)	
**Cardiovascular Diseases**					<0.001
Yes	376 (22.7)	614 (24.7)	2,683 (21.2)	893 (30.2)	
**Respiratory Diseases**					<0.001
Yes	128 (7.7)	64 (2.6)	877 (6.9)	249 (8.4)	
**Digestive Diseases**					<0.001
Yes	114 (6.9)	188 (7.6)	905 (7.2)	236 (8.0)	
**Genitourinary Diseases**					<0.001
Yes	286 (17.3)	290 (11.6)	1,335 (10.6)	423 (14.3)	
**Skin Diseases**					<0.001
Yes	18 (1.1)	32 (1.3)	123 (1.0)	34 (1.1)	
**Musculoskeletal Diseases**					<0.001
Yes	113 (6.8)	181 (7.3)	920 (7.3)	218 (7.4)	
**Mortality**					<0.001
Dead	593 (35.8)	999 (40.2)	4,928 (39.0)	1,446 (48.9)	

NHB, non-Hispanic Black; NHW, non-Hispanic White; SEER, Surveillance, Epidemiology, and End Results Program; U.S., United States.

### 3.4 Breast cancer treatment

In terms of treatment by subtype, **Supplementary Table S2** shows that patients with the Luminal A (27.7%) and Luminal B (22.7%) subtypes of breast cancer received the highest rates of hormone therapy, compared to HER2 (2.7%) and TNBC (1.7%) subtypes, who generally do not qualify for hormone therapy. Over half of patients with HER2 (51.3%), TNBC (49.0%), and Luminal B (45.0%) subtypes received chemotherapy, whereas <20% of patients across all subtypes received radiation therapy. Mastectomies were performed with a high percentage in all patients across all subtypes, with 66.8% for Luminal A, 60.7% for Luminal B, 60.5% for TNBC, and 57.4% for HER2. Distal surgeries were only performed in 2–3% of patients across all BC subtypes.

We next compared BC treatment based on race, ethnicity, and birthplace (**Table 3**). The use of hormone therapy was higher among NHW and Hispanics born in Mexico (26.1% and 23.4%) compared with Hispanics born in the U.S. and NHB (21.5% and 21.0%). Hispanics born in Mexico had the highest rates of chemotherapy (47.3%), followed by NHB (40.0%), Hispanics born in the U.S. (37.9%), and NHW (30.6%). NHW patients had the highest rates of radiation therapy (21.7%), whereas NHB patients had the lowest rates of radiation therapy (18.1%). Lower rates of mastectomies were observed for NHB patients (50.2%) compared with 55–63% for Hispanic and NHW (**Table 3**). However, patients who did not have a mastectomy may have received other forms of surgical intervention.

### 3.5 Unadjusted effects of covariates on breast cancer mortality

**Figure 2:**
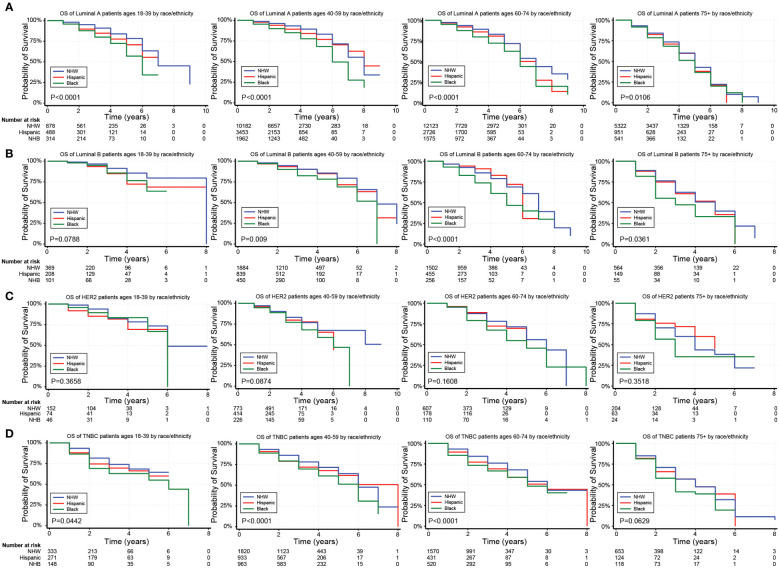
Overall survival (OS) for the different BC subtypes based on race/ethnicity and stratified by age. **(A–D)** Kaplan Meier curves show the OS for patients diagnosed with the Luminal A **(A)**, Luminal B **(B)**, HER2 **(C)**, and TNBC **(D)** subtypes of BC based on race and ethnicity. All subtypes were stratified by the following age groups from left to right: 18–39 years, 40–59 years, 60–74 years, and 75+ years.

**Figure 3:**
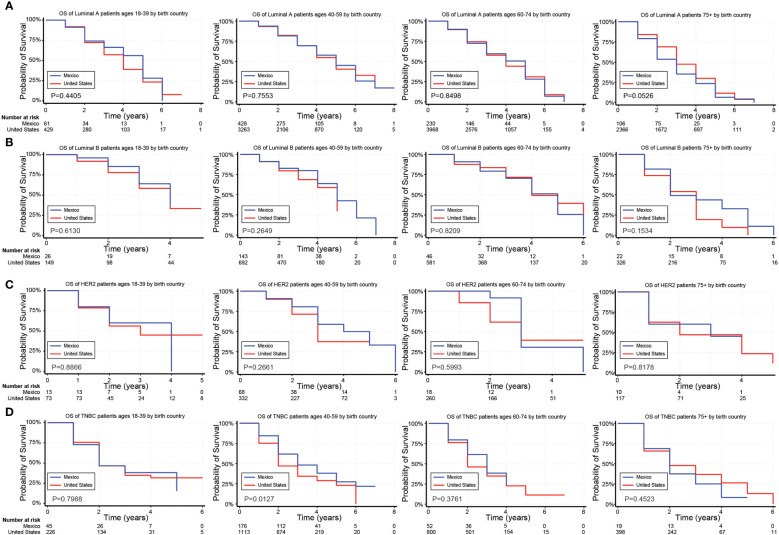
Overall survival (OS) for Hispanic BC patients based on birthplace and stratified by age. **(A–D)** Kaplan Meier curves show the OS for Hispanic patients diagnosed with the Luminal A **(A)**, Luminal B **(B)**, HER2 **(C)**, and TNBC **(D)** subtypes of BC based on birthplace. All subtypes were stratified by the following age groups from left to right: 18–39 years, 40–59 years, 60–74 years, and 75+ years.

**Figure 4:**
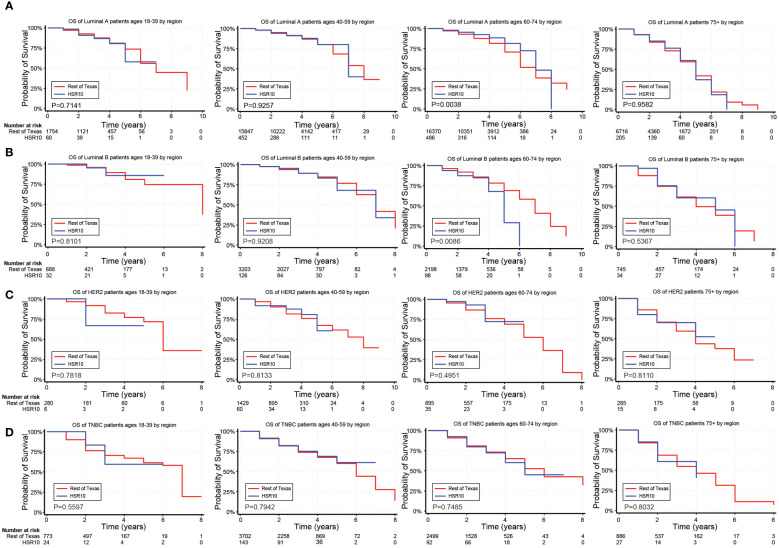
Overall survival (OS) for BC patients based on geographic location near the U.S./Mexico border. **(A–D)** Kaplan Meier curves show the OS for patients diagnosed with the Luminal A **(A)**, Luminal B **(B)**, HER2 **(C)**, and TNBC **(D)** subtypes of BC based on geographic location near the U.S./Mexico border (HSR10). All subtypes were stratified by the following age groups from left to right: 18–39 years, 40–59 years, 60–74 years, and 75+ years.

In unadjusted analyses, the outcomes for Hispanic BC patients initially appeared better than those for non-Hispanics diagnosed with the Luminal A and TNBC subtypes (**Supplementary Table S4**). Black race, on the other hand, was associated with worse outcomes for BC patients diagnosed with the Luminal A, Luminal B, and TNBC subtypes (**Supplementary Table S4**). **Figures 2**–**4** depict the OS by BC subtype based on race/ethnicity, birthplace, and geographic location, segmented by age. In the Luminal A subtype of BC, worse survival outcomes were observed in every age category associated with race/ethnicity (**Figure 2A**). In contrast, race had a more significant impact than ethnicity in the Luminal B subtype (**Figure 2B**). Race/ethnicity did not affect survival in the HER2 subtype of BC (**Figure 2C**), whereas it negatively affected OS in the younger TNBC patients (**Figure 2D**). Birthplace had little effect on OS in any BC subtype (**Figures 3A–D**), except for TNBC patients aged 40–59 years, where Hispanic patients born in Mexico demonstrated better OS than those born in the U.S. (**Figure 3D**). Lastly, residence in HSR10 near the U.S./Mexico border had little impact on OS (**Figures 4A–D**), except for Luminal A and Luminal B patients aged 60–74 years. Interestingly, patients in this category residing in HSR10 have better OS than the rest of Texas for Luminal A patients (**Figure 4A**) but worse for Luminal B patients (**Figure 4B**). These data underscore the importance of subtype, birthplace, and age in analyzing the effect of race, ethnicity, and geographic location on BC outcomes.

### 3.6 Adjusted effects of covariates on breast cancer mortality

***Luminal A.*** For patients with the Luminal A subtype of BC, NHB patients had an 18% increase in mortality (p<.0001). Hispanic ethnicity had a similar effect, regardless of birthplace. Residence near the U.S./Mexico border (HSR10), on the other hand, had no significant impact on mortality compared with residence in other areas of Texas (p=0.066). Comorbidities were associated with worse outcomes for patients with the Luminal A subtype of BC. In contrast, hormone therapy, radiation therapy, mastectomy, and urbanization were associated with better survival outcomes (**Table 4**).

**Table 4:** Factors contributing to the overall survival (OS) of sub-cohort 2 in multivariate Cox regression analyses.

Subtype Multivariable (Sub-Cohort 2)	Luminal A		Luminal B		HER2		TNBC	
	HR* & CI	P	HR* & CI	P	HR* & CI	P	HR* & CI	P
Age Ranges								
18-39	(ref)	---	(ref)	---	(ref)	---	(ref)	---
40-59	0.82 (0.68-1.00)	**0.047**	0.94 (0.70-1.27)	0.693	1.12 (0.70-1.79)	0.631	0.85 (0.71-1.03)	0.104
60-74	1.19 (0.99-1.44)	0.066	1.38 (1.01-1.89)	**0.042**	1.28 (0.80-2.05)	0.308	0.95 (0.78-1.15)	0.573
75+	2.03 (1.68-2.46)	**<.0001**	2.53 (1.82-3.52)	**<.0001**	2.37 (1.40-4.01)	**<.0001**	1.10 (0.87-1.39)	0.430
Ethnicity/Race Country of Birth								
NHW USA	(ref)	---	(ref)	---	(ref)	---	(ref)	---
Hispanic Mexico	1.18 (1.00-1.38)	**0.049**	0.86 (0.64-1.17)	0.339	0.85 (0.53-1.37)	0.511	1.03 (0.83-1.27)	0.821
Hispanic USA	1.17 (1.05-1.30)	**0.006**	1.06 (0.85-1.33)	0.603	1.13 (0.78-1.63)	0.524	1.25 (1.06-1.46)	**0.006**
NHB USA	1.18 (1.09-1.27)	**<.0001**	0.99 (0.79-1.24)	0.921	1.56 (1.17-2.09)	**0.003**	1.10 (0.97-1.26)	0.146
HSR								
Rest of Texas	(ref)	---	(ref)	---	(ref)	---	(ref)	---
HSR 10	0.80 (0.64-1.01)	0.066	0.95 (0.65-1.39)	0.790	1.49 (0.81-2.71)	0.197	0.83 (0.59-1.15)	0.258
Hormone Therapy								
No	(ref)	---	(ref)	---	(ref)	---	(ref)	---
Yes	0.86 (0.80-0.93)	**<.0001**	0.81 (0.68-0.96)	**0.014**	0.73 (0.34-1.53)	0.403	1.15 (0.86-1.53)	0.352
Chemotherapy								
No	(ref)	---	(ref)	---	(ref)	---	(ref)	---
Yes	1.05 (0.96-1.15)	0.267	0.79 (0.66-0.94)	**0.009**	0.854 (0.64-1.13)	0.275	0.76 (0.67-0.87)	**<.0001**
Radiation Therapy								
No	(ref)	---	(ref)	---	(ref)	---	(ref)	---
Yes	0.87 (0.79-0.95)	**0.002**	0.93 (0.77-1.13)	0.471	0.89 (0.68-1.17)	0.396	0.96 (0.84-1.10)	0.547
Mastectomy								
No	(ref)	---	(ref)	---	(ref)	---	(ref)	---
Yes	0.63 (0.57-0.69)	**<.0001**	0.64 (0.52-0.79)	**<.0001**	0.73 (0.55-0.95)	**0.022**	0.70 (0.61-0.80)	**<.0001**
Distal Lymph or Tissue/Organ Surgery								
No	(ref)	---	(ref)	---	(ref)	---	(ref)	---
Yes	1.00 (0.83-1.20)	0.990	0.71 (0.43-1.17)	0.181	0.91 (0.50-1.67)	0.766	0.90 (0.67-1.22)	0.503
Urbanization								
Rural	(ref)	---	(ref)	---	(ref)	---	(ref)	---
Semi-Urban	0.89 (0.81-0.98)	**0.016**	1.02 (0.81-1.28)	0.863	1.08 (0.73-1.58)	0.714	1.09 (0.91-1.30)	0.347
Number of Comorbidities								
None	(ref)	---	(ref)	---	(ref)	---	(ref)	---
1-2	0.92 (0.84-1.02)	0.101	0.93 (0.75-1.15)	0.502	1.22 (0.92-1.60)	0.165	1.01 (0.88-1.17)	0.885
3-4	1.01 (0.90-1.12)	0.905	0.97 (0.76-1.24)	0.839	0.83 (0.57-1.19)	0.305	0.94 (0.80-1.11)	0.481
5-6	1.16 (1.03-1.32)	**0.016**	1.36 (1.05-1.76)	**0.020**	1.45 (0.83-2.51)	0.189	1.08 (0.90-1.31)	0.404
7-8	1.31 (1.12-1.53)	**<.0001**	1.26 (0.90-1.75)	0.178	1.36 (0.83-2.24)	0.222	0.95 (0.74-1.22)	0.691
9-10	1.37 (1.17-1.61)	**<.0001**	1.08 (0.72-1.63)	**0.711**	1.68 (0.92-3.05)	0.090	1.11 (0.86-1.45)	0.425

CI, confidence interval; HER2, human epidermal growth factor receptor 2; HR, hazard ratio; HSR, health service region; NHB, non-Hispanic Black; NHW, non-Hispanic White; SEER, Surveillance Epidemiology and End Results; TNBC, triple-negative breast cancer. * HR reported after adjusting for the remaining variables included in the table.

Bold numbers represent statistically significant p values.

***Luminal B.*** For patients with the Luminal B subtype of BC, race, ethnicity, and geographic location did not affect mortality rates. Regional or distant metastases and comorbidities were associated with worse outcomes for patients with the Luminal B subtype of BC, whereas hormone therapy, chemotherapy, and mastectomy were associated with better outcomes (**Table 4**).

***HER2.*** Mortality risk increased by >2-fold in patients aged 75 years and older (p<0.0001). We observed no differences in mortality comparing patients by ethnicity, birthplace, and residency. However, NHB patients had a 56% increase in mortality risk compared with NHWs (p=0.003). Regional and distant metastasis correlated with worse outcomes, whereas mastectomy significantly reduced mortality risk for patients with HER2+ BC (**Table 4**).

***TNBC*.** Age did not affect mortality risk in the most aggressive TNBC subtype. While race did not affect mortality risk in TNBC, Hispanic patients from the U.S. had a 25% increase in mortality risk compared with NHW patients (p=0.006). Birthplace in Mexico, on the other hand, had no significant effect. Regional and distant metastasis correlated with worse outcomes for TNBC patients, whereas chemotherapy and mastectomy were associated with better outcomes (**Table 4**).

## 4 Discussion

Prior work has demonstrated that racial disparities differ by tumor subtype (18, 56, 57). Our data confirmed our hypothesis that the effect of race/ethnicity on breast cancer outcomes depends on the BC subtype and country of origin but not geographic location near the U.S./Mexico border. Ethnic and racial disparities in cancer outcomes, particularly breast cancer, persist despite progress in understanding contributing factors (58,) (59). This is especially true for cancer patients living near the U.S./Mexico border (25, 26, 60). The Hispanic population is the largest minority group in the U.S., representing >18% of the population (61). Hispanic/Latinx refers to individuals with Latin American roots, erroneously generalized to a single homogenous group due to commonalities in language, values, and migration history (61). This study focuses on BC disparities among Hispanic populations, emphasizing the need to analyze subgroups separately due to diverse biological, behavioral, sociocultural, and socioeconomic factors. Our investigation compared BC presentation and outcomes for U.S.-born and Mexico-born Hispanic patients compared with NHW and NHB populations. We also examined the effects of border proximity on BC outcomes and analyzed the impact of various treatment interventions on OS. Our findings revealed worse outcomes for U.S.-born Hispanic patients with TNBC compared with all other racial and ethnic groups. In contrast, U.S.-born Hispanic, Mexico-born Hispanic, and NHB patients with the Luminal A subtype had worse outcomes compared with NHW patients. Surprisingly, proximity to the U.S./Mexico border improved OS for Luminal A patients aged 60–74 years but reduced OS for Luminal B patients aged 60–74 years. Understanding factors that influence the presentation and survival of BC patients is crucial for prioritizing resources, addressing systemic deficiencies, and developing targeted approaches for prevention, screening, treatment, and adherence within these high-risk communities.

Individuals living in the border region face many challenges, such as lower median income, higher poverty rates, and reduced educational attainment leading to lower utilization of screening services (62, 63). Hispanic women with lower educational attainment have difficulty communicating with healthcare providers and may view BC as a terminal disease, unaware of effective treatment options (64). Patients in this region are more likely to be uninsured, and Hispanic patients are more frequently covered by Medicaid than NHWs (62). Previous studies have noted that Hispanics are more likely to be diagnosed with aggressive subtypes of BC, such as TNBC, and present at more advanced stages than NHWs (20). However, differences in survival for Hispanic patients are only partially explained by tumor characteristics. Low SES is linked to increased mortality due to financial concerns, lack of insurance coverage, and limited access to care and screening services (20).

Despite these challenges, some studies have reported lower BC incidence rates and better survival among Hispanics, underscoring the complexity of these disparities (27). The influence of acculturation on higher BC presentation and mortality may reflect the adoption of unhealthy behaviors (such as poor diet and smoking) by those who have been in the U.S. for a more extended period of time. Still, it may also be due to self-selection, where unacculturated immigrants are healthy when they enter the U.S. and may leave the country if diagnosed with cancer and become lost to follow-up (8, 27). This finding may be due to disparities in insurance coverage, as Hispanics are more likely to be underinsured, resulting in problems with healthcare access (65).

Delays in treatment and adherence issues also affect survival rates (58, 66). Hispanics are more likely to experience delayed initiation of chemotherapy and longer wait times (over 30 days) for surgery than NHWs (58, 66). Possible reasons for delays in treatment include SES, insurance status, problems accessing care, and geographic distance from cancer centers (58, 66). Additionally, poor adherence to treatment leads to cancer relapse, higher rates of hospitalization, and shorter OS (67). A survey of Hispanics in the border region found medium-to-low compliance with long-term adjuvant endocrine therapy (67). Frequently cited reasons were forgetfulness, avoidance of medication side effects, and high cost, but educational programs and frequent reassessment were identified as potential strategies for improvement (67). Smaller sample sizes of patients in HSR10 and loss to follow-up, primarily ages 18–39, may affect the interpretation of results. Further investigation is required to identify additional factors affecting OS for aggressive BC subtypes.

In our study, NHB and Hispanic patients in Texas were diagnosed at younger ages than NHW patients for all diseases analyzed (P<0.0001), consistent with previous reports (68, 69). NHB, U.S.-born Hispanic, and Mexico-born Hispanic patients had worse outcomes in the Luminal A subtype, indirectly supporting the potential role of genetic factors associated with Indigenous and African American ancestry in South American populations (70). Notably, BC patients aged 40–59 with the Luminal A subtype showed better OS, possibly due to tissue maturation after breastfeeding (71). Patients 40–59 years old have most likely had one or more children and a longer duration of breastfeeding compared with younger patients and most likely accumulated fewer comorbidities than older patients (71). Foreign-born women also exhibit lower BC mortality rates, often attributed to fewer risk factors like obesity, hormone replacement therapy, alcohol consumption, and a sedentary lifestyle (72). For example, a study analyzing BC mortality rates of women residing in California found that women born in Mexico had a 28% lower mortality rate than those born in the U.S. (12.9 versus 18.0 per 100,000, respectively) (73–75). In our analysis, race remains a significant risk factor, with Black patients experiencing a higher risk of death across all BC subtypes (76).

A major strength of our population-based study is a comprehensive focus on Mexican Hispanic populations by birthplace. Additionally, we included multiple BC subtypes based on the effects of ethnicity, race, border proximity, and age (52). We did not control for the stage at diagnosis, since it is most likely on the causal path between race, ethnicity, and OS (52). Controlling for causal intermediates using standard statistical methods will lead to biased estimates of the total effect (52, 77). Limitations to our study include using a single-state registry without critical behavioral and genomic data and the large number of exclusions based on our stringent eligibility criteria (i.e., lack of BC subtype or ethnicity information). While time to treatment between Hispanic and non-Hispanic patients was another potential limitation, the analysis was only possible with extensive data loss due to irregularities with the reporting of dates within the dataset, preventing us from calculating the time from diagnosis to initiation of treatment.

Greater racial and ethnic diversity in biobanks and genome-wide association studies will allow for improved risk stratification and clinical decision-making (78, 79). Future studies should focus on enhancing diversity, equity, inclusion, and accessibility in healthcare, as well as increased clinical trial participation from diverse backgrounds, to address the needs of at-risk communities (80). On top of that, future studies should examine the effects of various treatment protocols and adherence rates on relapse rate and OS across different racial/ethnic groups. Furthermore, to establish a clear understanding of the socioeconomic factors that impact the pathways leading to breast cancer in minority women, it is essential to conduct genetic, epigenomic, and transcriptomic studies, which may provide novel approaches for diagnosing and treating breast cancer among vulnerable populations. Increasing minority participation in clinical trials will be critical for learning the pathophysiological aspects of treatment. Finally, immediate caregivers, such as nurses and family members, significantly impact treatment outcomes. Therefore, enhancing diversity among healthcare providers would be highly beneficial.

## Data Availability

The original contributions presented in the study are included in the article/**Supplementary Material**. Further inquiries can be directed to the corresponding authors.
