# Supplementary material for: Racial/ethnic differences in the clinical presentation and survival of breast cancer by subtype
Source: Front Oncol. 2024 Aug 16;14:1443399. doi: 10.3389/fonc.2024.1443399 (PMC11361935; doi:10.3389/fonc.2024.1443399)
Supplement: Supplementary file 1 [file Table1.docx]

Supplementary Material

Racial/Ethnic Differences in the Clinical Presentation and Survival of Breast Cancer by Subtype

**Vutha Nhim^1,2^, Alfonso E. Bencomo-Alvarez^3,4^, Luis Alvarado^5^, Michelle Kilcoyne^1,6^, Mayra A. Gonzalez-Henry^3^, Idaly M. Olivas^3^, Mehrshad Keivan^7^, Sumit Gaur^8^, Zuber D. Mulla^9-11^, Alok K. Dwivedi^1,5^, Shrikanth S. Gadad^1,3^* & Anna M. Eiring^1,3^***

^1^Paul L. Foster School of Medicine, Texas Tech University Health Sciences Center El Paso, El Paso, TX, USA

^2^UAMS Washington Regional Medical Center, Fayetteville, AR, USA

^3^Center of Emphasis in Cancer, Department of Molecular and Translational Medicine, Texas Tech University Health Sciences Center El Paso, El Paso, TX, USA

^4^St. Jude Children’s Research Hospital, Memphis, TN, USA

^5^Biostatistics and Epidemiology Consulting Lab, Office of Research, Texas Tech University Health Sciences Center El Paso, El Paso, TX, USA

^6^Baylor College of Medicine, Houston, TX, USA

^7^Burrell College of Osteopathic Medicine, University Park, NM, USA

^8^Department of Internal Medicine, Texas Tech University Health Sciences Center El Paso, El Paso, TX, USA

^9^Department of Obstetrics and Gynecology, Texas Tech University Health Sciences Center El Paso, El Paso, TX, USA

^10^Office of Faculty Development, Texas Tech University Health Sciences Center El Paso, El Paso, TX, USA

^11^Julia Jones Matthews School of Population and Public Health, Texas Tech University Health Sciences Center, Abilene, TX, USA

*** Correspondence:**Shrikanth S. Gadad, Ph.D.: [shrikanth.gadad@ttuhsc.edu](mailto:shrikanth.gadad@ttuhsc.edu)

Anna M. Eiring, Ph.D.: [anna.eiring@ttuhsc.edu](mailto:anna.eiring@ttuhsc.edu).

Keywords: Race/ethnicity, Breast cancer (BC), United States/Mexico border, Population-based study, Cancer health disparities.

**Supplementary Tables**

| **Patient Characteristics** | **Excluded** |
| --- | --- |
| **N (%)** | **213,283 (100)** |
| **Age Ranges** |  |
| 18-39 | 13,684 (6.4) |
| 40-59 | 90,016 (42.2) |
| 60-74 | 70,659 (33.1) |
| 75+ | 38,924 (18.3) |
| **Race & Ethnicity** |  |
| Hispanic | 35,941 (17.0) |
| NHW | 149,324 (70.0) |
| NHB | 22943 (11.0) |
| **HSR** |  |
| Rest of Texas | 207,249 (97.2) |
| HSR 10 | 6,034 (2.8) |
| **Country of Birth** |  |
| U.S. | 93,343 (43.8) |
| Mexico | 5,201 (2.4) |
| **Ethnicity/Race & Birthplace** |  |
| Hispanic Born in U.S. | 11,655 (5.5) |
| Hispanic Born in Mexico | 5,028 (2.4) |
| NHW Born in U.S. | 68,867 (32.3) |
| NHB Born in U.S. | 12,106 (5.7) |
| Hispanic Other | 19,258 (9.0) |
| Non-Hispanic Other | 91,294 (42.8) |
| **Ethnicity/Race HSR** |  |
| Hispanic HSR 10 | 4,094 (1.9) |
| Hispanic - Rest of Texas | 31,847 (14.9) |
| Non-Hispanic | 172,267 (80.8) |
| **Urbanization** |  |
| Rural | 2,332 (1.1) |
| Semi-Urban | 28,310 (13.3) |
| Urban | 182,616 (85.6) |
| **Primary Insurance** |  |
| Uninsured | 4,122 (1.9) |
| Private Insurance | 32,984 (15.5) |
| Medicaid/Medicare | 28,809 (13.5) |
| **Poverty Level** |  |
| No | 160,081 (75.1) |
| Yes | 52,816 (24.8) |

**Table S1. Patient characteristics of the excluded cohort (213,283 breast cancer patients from the Texas Cancer Registry from 1995-2016) who did not have complete records on BC subtype.** HSR, health service region; NHB, non-Hispanic Black; NHW, non-Hispanic White; U.S., United States.

**
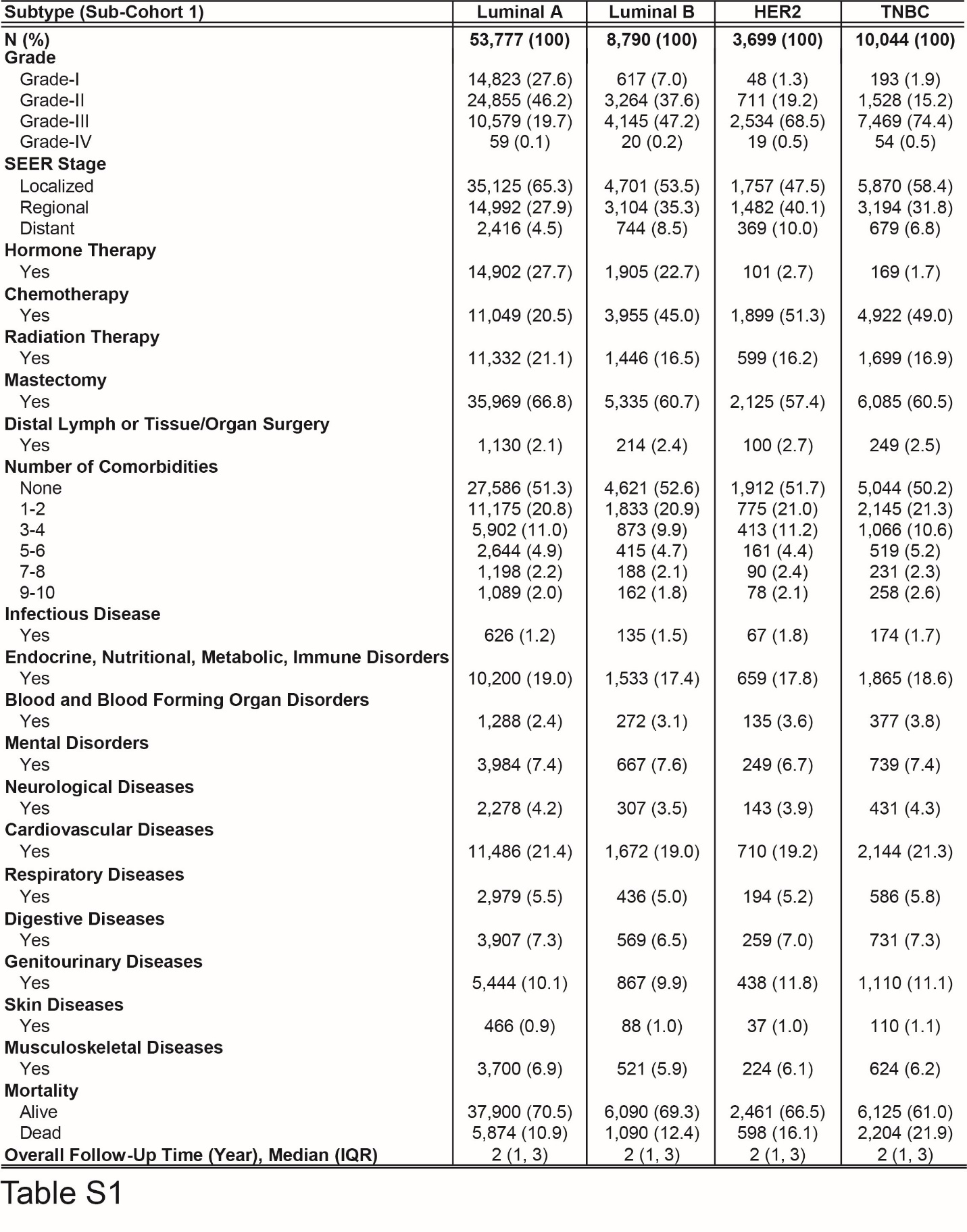
Table S2. Breast cancer (BC) patient distribution of sub-cohort 1 (76,310 BC patients from the Texas Cancer Registry from 1995-2016) according to subtype, based on tumor grade, treatment, and comorbidity burden.** HER2, human epidermal growth factor receptor 2; TNBC, triple-negative breast cancer.

| **Ethnicity (Sub-Cohort 2)** | **Mexico-Born**  **Hispanic** | **U.S.-Born Hispanic** | **NHW** | **NHB** | **p-value** |
| --- | --- | --- | --- | --- | --- |
| **N (%)** | **1,656 (100)** | **2,484 (100)** | **12,650 (100)** | **2,958 (100)** |  |
| **Age Ranges** |  |  |  |  | <0.001 |
| 18-39 | 162 (9.8) | 237 (9.5) | 527 (4.2) | 226 (7.6) |  |
| 40-59 | 924 (55.8) | 1,020 (41.0) | 4,041 (31.9) | 1,321 (44.7) |  |
| 60-74 | 395 (23.9) | 839 (33.8) | 5,049 (39.9) | 1,006 (34.0) |  |
| 75+ | 175 (10.6) | 388 (15.6) | 3,033 (24.0) | 405 (13.7) |  |
| **HSR** |  |  |  |  | <0.001 |
| Rest of Texas | 1,254 (75.7) | 2,253 (90.7) | 12,543 (99.2) | 2,942 (99.5) |  |
| HSR 10 | 402 (24.3) | 231 (9.3) | 107 (0.8) | 16 (0.5) |  |
| **Urbanization** |  |  |  |  | <0.001 |
| Rural | 1 (0.0006) | 15 (0.6) | 201 (1.6) | 15 (0.5) |  |
| Semi-Urban | 89 (5.4) | 266 (10.7) | 1,849 (14.6) | 202 (6.8) |  |
| Urban | 1,566 (94.6) | 2,202 (88.6) | 10,599 (83.8) | 2,740 (92.6) |  |
| **Primary Insurance** |  |  |  |  | <0.001 |
| Uninsured | 707 (42.7) | 273 (11.0) | 434 (3.4) | 408 (13.8) |  |
| Private Insurance | 294 (17.8) | 940 (37.8) | 5,365 (42.4) | 961 (32.5) |  |
| Medicaid/Medicare | 529 (31.9) | 1,134 (45.7) | 6,286 (49.7) | 1,440 (48.7) |  |
| **Poverty Level** |  |  |  |  | <0.001 |
| Yes | 883 (53.3) | 1,050 (42.3) | 1,940 (15.3) | 1,269 (42.9) |  |

**Table S3. Patient distribution of sub-cohort 2 (19,748 breast cancer [BC] patients with complete data on subtype/race/ethnicity/birthplace/age) according to BC subtype, based on race, ethnicity, birthplace, and poverty indicators.** HSR, health service region; NHB, non-Hispanic Black; NHW, non-Hispanic White; U.S., United States.


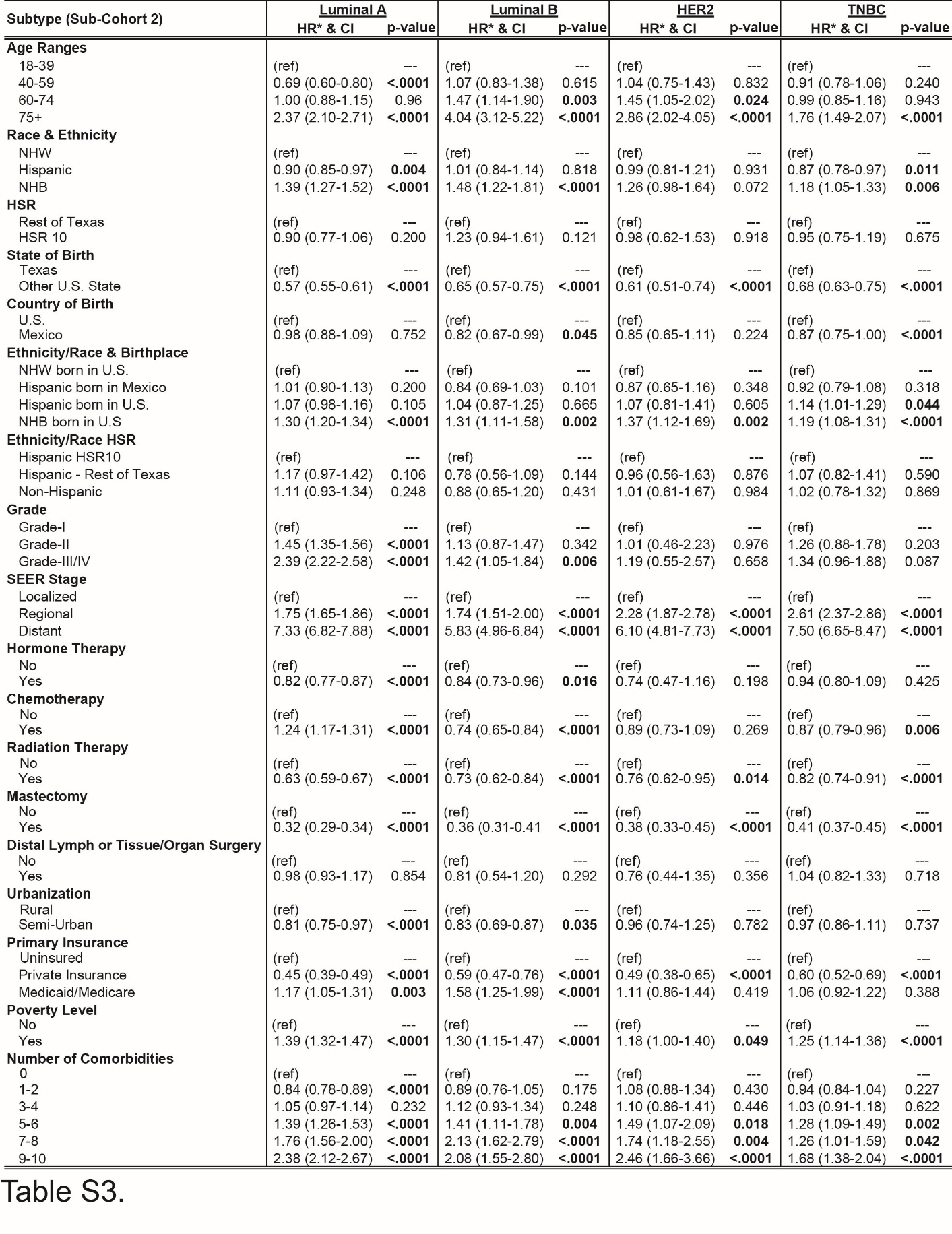


**Table S4. Factors contributing to the overall survival (OS) of sub-cohort 2 in univariate Cox regression analyses.** HER2, human epidermal growth factor receptor 2; HSR, health service region; NHB, non-Hispanic Black; NHW, non-Hispanic White; SEER, Surveillance Epidemiology and End Results; TNBC, triple-negative breast cancer.
